# Development of the Ontogenetic Self-Regulation Clock

**DOI:** 10.3390/ijms23020993

**Published:** 2022-01-17

**Authors:** Sari Goldstein Ferber, Aron Weller, Michal Ben-Shachar, Gil Klinger, Ronny Geva

**Affiliations:** 1 Department of Psychology, Bar Ilan University, Ramat Gan 5290002, Israel; Aron.Weller@biu.ac.il (A.W.); Ronny.Geva@biu.ac.il (R.G.); 2 The Leslie and Susan Gonda (Goldschmied) Multidisciplinary Brain Research Center, Bar Ilan University, Ramat Gan 5290002, Israel; Michal.Ben-Shachar@biu.ac.il; 3 Department of Neonatology, Schneider Children’s Medical Center, Sackler Medical School, Tel Aviv University, Petach Tikvah 4920235, Israel; gilkl@tauex.tau.ac.il

**Keywords:** self-regulation, corticospinal tract, autonomic nervous system, heart rate variability, EEG, white matter, excitation-inhibition

## Abstract

To date, there is no overarching proposition for the ontogenetic-neurobiological basis of self-regulation. This paper suggests that the balanced self-regulatory reaction of the fetus, newborn and infant is based on a complex mechanism starting from early brainstem development and continuing to progressive control of the cortex over the brainstem. It is suggested that this balance occurs through the synchronous reactivity between the sympathetic and parasympathetic systems, both which originate from the brainstem. The paper presents an evidence-based approach in which molecular excitation-inhibition balance, interchanges between excitatory and inhibitory roles of neurotransmitters as well as cardiovascular and white matter development across gestational ages, are shown to create sympathetic-parasympathetic synchrony, including the postnatal development of electroencephalogram waves and vagal tone. These occur in developmental milestones detectable in the same time windows (sensitive periods of development) within a convergent systematic progress. This ontogenetic stepwise process is termed “the self-regulation clock” and suggest that this clock is located in the largest connection between the brainstem and the cortex, the corticospinal tract. This novel evidence-based new theory paves the way towards more accurate hypotheses and complex studies of self-regulation and its biological basis, as well as pointing to time windows for interventions in preterm infants. The paper also describes the developing indirect signaling between the suprachiasmatic nucleus and the corticospinal tract. Finally, the paper proposes novel hypotheses for molecular, structural and functional investigation of the “clock” circuitry, including its associations with other biological clocks. This complex circuitry is suggested to be responsible for the developing self-regulatory functions and their neurobehavioral correlates.

## 1. Introduction

There are sparse data on the stepwise process of autonomic nervous system (ANS) maturation and its coupling with cortical development. This is especially rare regarding fetal and postnatal development. The available data on ANS coupling with the cortex focus on later life, e.g., [1], highlighting the timely convergent perspective presented in this paper. Specifically, the paper aims to show that there are convergent trajectories for the gradual development of the ANS in fetuses and infants, emphasizing the essential role of both branches of the ANS, the sympathetic and parasympathetic systems, as well as the role of the progressive development of the brainstem in the development of these two branches from early stages of gestation onwards. This paper suggests a new evidence-based theory on the ontogenetic development of self-regulation.

The interplay between the two systems of the ANS, components that affect or delay the establishment of the synchrony between the two systems, as well as the developmental time windows, has been neglected, although implicated, in general, in the theoretical concept of self-regulation. 

As arising from integration of published data, this interplay includes synchronous occurrence, which reaches detectable time windows along its progress during embryonic, fetal and neonatal life. As both the sympathetic and parasympathetic systems originate in the brainstem, the brainstem has a crucial role in the emergence of synchronized control of self-regulation. In this paper, the focus is on the brainstem’s developmental impact on both the ANS and the developing cortex as related to the emergence of self-regulation. The suggestion is that ANS maturation occurs within the developing control of the cortex over the brainstem through particular neurotransmitter interchange originating in the brainstem. 

Although in our view, the process of ANS maturation starts early during gestation, this process is enhanced following the penetration of the cortical plate by thalamocortical afferents during mid-gestation, the same time window (sensitive period) for many other developing processes (to be further described below). According to this view, self-regulation depends on the developing ANS synchrony to support later resolution of the subcortical plate following advancement of the thalamic fibers into the cortical plate and its further developmental sensorimotor progress [2,3]. 

Here, a neuro-anatomical perspective for cortical-brainstem connectivity through the corticospinal tract (CST) is also presented. This developmental process is described as dependent on molecular, structural and connectivity\functional aspects of the neuronal cell, its neural network wiring and the involved fiber myelination. It is further suggested that cortical coupling with the ANS is essential for brain inhibition-excitation balance within the developing organism and that the synchrony is resulting in required molecular and biochemical delicate conditions for accomplishing the interplay between the sympathetic and parasympathetic systems. 

Another suggestion is that ANS synchrony is achieved following cell migration from the brainstem to the cortex and the crucial interchange of neurotransmitters affecting proliferation, differentiation and the onset of myelination processes, and is intensified at the time window of mid-gestation through the first two years of life. This time window has been identified as a period of excitation-inhibition development, which results in a mature balance and parallels the neurobehavioral conceptualization of self-regulation. An additional suggestion is that the development of this ANS synchrony is programmed early in life and that it depends on brainstem maturity. It is moreover proposed that this process follows an ontogenetic clock in term born infants. Based on the theoretical concept of intra-individual self-regulation, which has been studied earlier [4,5,6], it is suggested that the term “self-regulation clock” describes the developmental milestones of ANS synchrony from gestation to the first two years of postnatal life. The current postulates are based on neuroanatomical, neurochemical, electroencephalogram (EEG) and Magnetic Resonance Imaging (MRI) data as well as on vagal tone measures, cardiovascular development and connectivity with the maturing cortex.

## 2. Self-Regulation: An Intra-Individual Perspective

There are two approaches to early life self-regulation: an intra-individual (infant) and inter-individual (infant self-regulation in the context of interactions with the caregivers), both of which address the regulatory difficulties of term and preterm infants, but from different angles [6,7,8,9,10,11]. The intra-individual approach’s relevance starts from gestation and the generation of the ANS in the fetus, while the inter-individual approach focuses on the measures of the child’s mutual interaction with his\her parental environment postnatally [12,13,14,15]. The current article focuses on the intra-individual approach both prenatally and postnatally. It focuses on the intra-individual self-regulation perspective, i.e., the potential strengths and thresholds of the growing fetus, preterm and term infant. We have demonstrated earlier the critical role of the parent for preterm infants during the critical time window of mid-gestation (e.g., [16]) as well as later in development for term born infants [16]. Thus, the intra-individual perspective of self-regulation does not exclude the well-accepted contribution of the sensitive parent to improved development of the child’s self-regulation. The intra-individual perspective details strengths and thresholds of the growing individual for improved orientation of the parents to these aspects of their child.

The unique view of the intra-individual approach lies in the focus on the infant rather than on the dyad (parent and infant), viewing him/her as a capsule that manages internal and external cues. It is applicable to the growing fetus in the womb with placental influences as well as to later stages of early development. This is a different focus on the growing individual, relating individuality to earlier stages of development than articulated to date. That is, the self-regulation of the growing individual within his\her own capsule is based on his\her physiological processes but is more than physiology per se from very early stages of development. 

**Figure 1:**
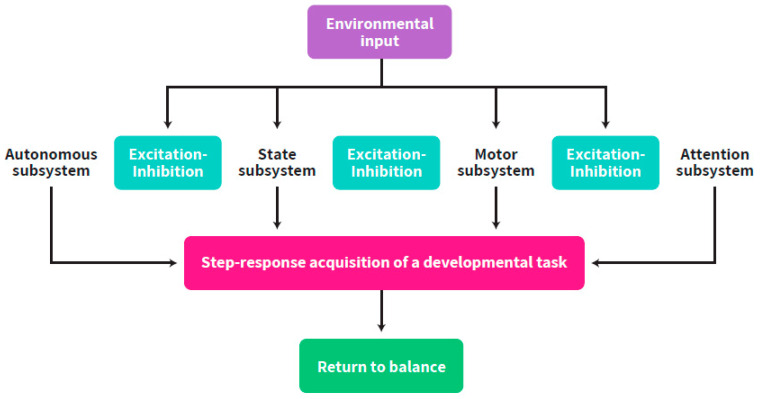
The interplay between the neurobehavioral subsystems through excitation-inhibition of each other while faced with environmental inputs and return to balance following achievement of a developmental task.

Regulation according to this approach has been defined [17,18] as the ability of the organism to return to baseline after mounting specific responses to external inputs. Intrapersonal neurobehavioral co-regulation is defined as the capacity of the organism to subordinate all neurobehavioral capacities that allows it to be adaptive to the environmental requirements including intra-uterine stimulation. It is also defined as the capacity of the organism to return to balance, following the adaptation of one enhanced neurobehavioral subsystem to the external inputs [18,19] and the acquisition of a new balance between all the subsystems. These processes support the successive maturation of all neurobehavioral subsystems, i.e., autonomic, motor, state and attentional [20,21], while working together, influencing each other [19], towards a coordinated increasingly differentiated balance by increasing or decreasing responses to each other’s state starting from early gestational age (GA). Self-regulation encompasses all neurobehavioral outcomes in the fetus, newborn and infant, which result from ANS level of balance (i.e., synchronized vs. non-synchronized) [9,11,22] (Figure 1).

**Figure 2:**
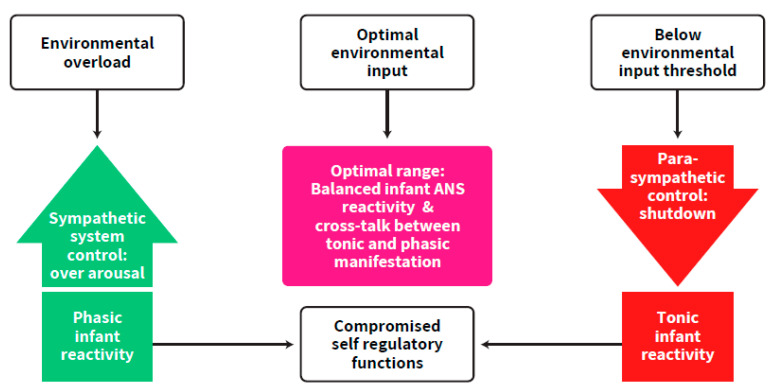
The imbalance and lack of synchrony in ANS functioning of the preterm infant compared to the optimal range. The ascending arrow describes conditions of over-arousal and sympathetic control. The descending arrow describes conditions of parasympathetic control up to shut-down. The other arrows describe the progress of the process in overloaded vs. under-stimulated conditions.

The importance of the parasympathetic system in regulating over-arousal as well as that of the sympathetic system in bringing the infant up to the arousal level needed for optimal developmental functioning have been discussed [6,9,23,24]. However, the focus on the synchrony between the two branches of the ANS as one working system aimed towards an ongoing balanced activation has not been adequately addressed. Our postulation follows a well-established concept of a required continuous balance between excitatory and inhibitory brain functions, suggesting that any deviation may put the individual at risk for adverse development [22]. Balanced activation, widely implied in the literature on the concept of self-regulation (e.g., [23,24,25], includes, according to our postulants, the balance between the two ANS branches and the excitation-inhibition balance between neurotransmitters. These types of balances are supposed to rescue the newborn from over-arousal and exhaustion as well as from systemic shutdown states [9,11,26] (Figure 2). It is emphasized here that this synchrony is a prerequisite for cortical maturation. To support this view, an author of this review, and colleagues, found that autonomic system measurements predicted Bayley mental scores in the first year of life, in term and preterm born infants [27].

Specifically, our studies show the interplay between the motor and the attention (sleep-wake states) neurobehavioral subsystems in full-term and in preterm infants (i.e., that motor subsystem activation is the source of inhibition of the attention system and vice versa) [19,28] and represent ANS synchrony and self-regulatory functions which are the prerequisite for acquisition of healthy successive developmental milestones.

**Figure 3:**
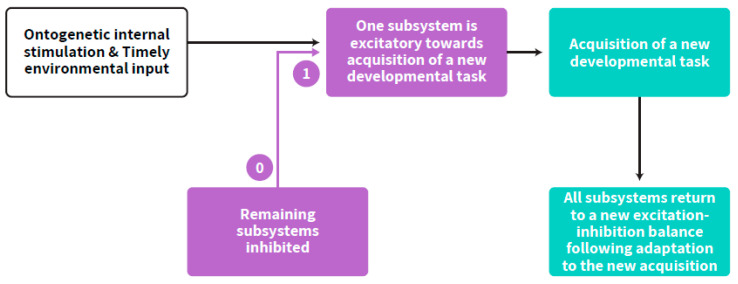
Step-response (text in yellow) acquisition of a developmental task running from zero (pre acquisition status) to one (acquired capacity, e.g., the first breath, the first step, the first word). The interplay between subsystems is a continuous process which is enhanced at an age-appropriate acquisition of any developmental task. The figure describes the process of a special pattern of interplay within the subsystems at such a momentary point of acquisition up to the return to a new balance and to advanced interplay of excitation-inhibition between all subsystems. The purple arrows represent the step-response. The other arrows represent the flow of the process.

Self-regulated acquisition of a new developmental stage from embryonic to postnatal early life, involves the temporal dominance of one neurobehavioral subsystem over the others [20,21] as well as time-limited interplay between more than one age-dependent subsystem [19], and therefore may be described with the term “step-response”, adapted from Control Theory in engineering (commonly used in the field of Neural Networks). The step response of a system in a given initial state consists of the time evolution of its outputs when its inputs are the source originating the step function. In control theory, step response is the time behavior of the output of a general system when its input changes from zero to one in a very short time [29]. It implies a developmental hierarchy according to age-appropriate neurobehavioral goals. Fluidity and interconnecting influences between all subsystems are maintained during the plateau part after achieving a certain developmental goal. The step response occurs all at once within an age-appropriate range for any specific developmental goal. Accordingly, adapting the approach from neural network theories, timely step-response acquisition of a new developmental task by the child, within the excitation-inhibition axis, follows a self-regulated neurobehavioral path [18]. The optimal amount of inhibition and excitation differs by developmental stage. Thus, the balance between excitation and inhibition is the crucial factor in development. This paper suggests that this balance is the essence of the developing ANS synchrony and its coupling with the cortex, which is the basis for the self-regulation clock (Figure 3).

Thus, our paper on the development of ANS synchrony in the brainstem-cortical circuits, its milestones during gestation, including mounting neurobehavioral reactions and return to balance, as well as conditions such as over arousal and shutdown, is timely. The developing control of the cortex over the brainstem represents the development of self-regulation as outlined in this review.

## 3. The Crucial Milestones of ANS Synchrony Development and the Multi-Level Consolidation of the Self-Regulation Clock

The progress of the developing synchrony between the sympathetic and parasympathetic systems involves interchanges in the excitatory vs. inhibitory role on the molecular level and the maturation of white matter structures. This process is detectable from early gestation through the age of two years of life. This part of the article will show the milestones of this process which consist of a crucial time window at mid-gestation, from molecular, neurotransmitter, neuroanatomical (as shown in MRI studies) and cortex connectivity (as shown in EEG studies) angles including cardiovascular development. Research shows that reformations along all these angles occur at the same time during gestation and early development, thus suggesting an essential ontogenetic clock of this development that has specific time windows for a stepwise development and specific trajectories along the same time points.

There are several pivotal processes affecting brain development such as proliferation, differentiation, myelination and cell migration. The main neurotransmitters involved in this progression will be considered, emphasizing the shift between excitation and inhibition that ends during mid-gestation (from 24 weeks GA) and the early postnatal period. Many of these neurotransmitters originate in the brainstem and affect development of the whole brain, including cortical maturation.

### 3.1. Early Gestation

Noradrenaline, the best marker of arousal and sympathetic activity, is concentrated in cells in the brainstem as early as five weeks of gestation, especially in the locus coeruleus within the caudal pons. Five major adrenergic tracts originate in the brainstem and innervate the whole brain. Adrenergic activity is among the strongest to initiate cell migration to the cortex and cortical neurogenesis [26]. At early gestation, the excitatory brain activity is based on noradrenergic [26] and cholinergic pathways [30] up to the stage of brainstem integrated development. Developmental studies showing a correlation between acetylcholine (Ach) receptor expression and the peak in Acetylcholinesterase (AchE) activity during periods of myelination, suggest ACh signaling as a mechanism for functional activity in the axon to stimulate myelination early in pregnancy [31]. In addition, glutamate receptors appear as early as week 8 in gestation [32].

**Figure 4:**
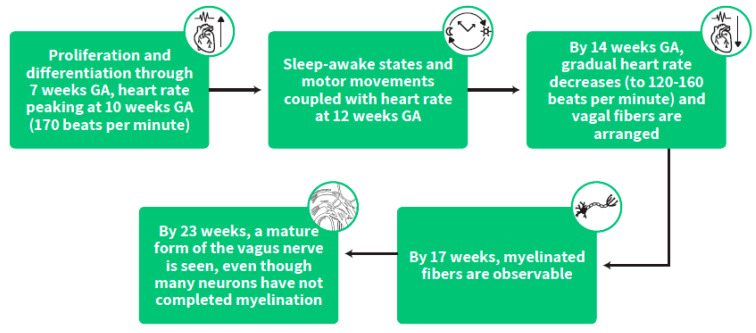
The stepwise stages of heart rate development and the timing of its coupling with fetal sleep states and behavior.

During this early stage of gestation, the heart is among the first organs to develop, undergoing proliferation and differentiation through 7 weeks GA with increasing heart rate peaking at 10 weeks GA (170 beats per minute) followed by a gradual decrease (to 120–160 beats per minute) from this peaking age onwards [33,34]. At this early time in life, vagal fibers are arranged, and by 14 weeks GA, the spiral development profile of myelination starts. By 17 weeks, myelinated fibers are observable and by 23 weeks a mature form of the vagus nerve is seen, even though many neurons have not completed myelination [35]. (Figure 4)

The functionality of the heart measured by heart rate, heart rate variability and vagal tone using ultrasonic tools during embryonic life is shown to correspond to neurobehavioral measures such as behavioral sleep-awake states and motor movements with a coupling onset of heart rate with those measures at 12 weeks GA [36]. Maturational changes in the innervation of sympathetic and parasympathetic autonomic processes [37], including vagal myelination [38], and alterations to central mediation within the medulla oblongata and adjacent loci [39] are among the most well-documented neural components that contribute to amplification of heart rate variability over gestation.

### 3.2. Mid- to Late-Gestation

The parasympathetic system undergoes accelerated maturation at 25–30 weeks GA [40,41]. Specifically, myelination of the vagus nerve increases from 24 weeks of gestation [38], suggesting the same timing for changes as those shown in excitatory and inhibitory pathways that involve the brainstem. Indeed, at this crucial timing, mid-gestation, ACh levels appear to be reduced and they turn to have an inhibitory role within the brainstem pathways [26,30]. A further reduction is evident following the transition from fetal to postnatal stages. This is shown by binding of muscarinic ACh receptors (mAChRs) [42]. In the developing brainstem, mAChRs localize to sites in which ACh via mAChR interactions has been shown in animal studies to influence regulatory functions [43,44,45,46].

Cells containing gamma-aminobutyric acid (GABA) are found in 20–40% of all neural terminals. GABA is regarded as inhibitory in the human brain. However, during early development it acts as a trophic factor that affects proliferation, migration, differentiation, synapse maturation and apoptosis [47,48]. C1- channels switch from depolarization to hyperpolarization during mid-gestation resulting in switching the GABAergic activity from excitatory to inhibitory. During this crucial time window, the K-Cl co-transporter KCC2 plays multiple roles in the physiology of central neurons, including influencing the development of neuronal circuits and the dynamic control of GABA and glycine signaling.

GABA-releasing synapses appear before glutamatergic synapses [49]. Glutamate transport is a primary mechanism for regulating extracellular levels of glutamate in the central nervous system [50]. In animal models, glutamate expression increased with age with a major reduction postnatally [50,51,52]. Thus, the well-established excitatory role of glutamate is evident during this crucial time window, as its levels increase from mid-gestation throughout fetal development at the late pregnancy stages, while the excitatory effects of ACh decrease. The GABAergic neuronal density increases in the human cortex over late gestation, also with a peak at term [53]. The second half of gestation is a period of rapid development of the cortical GABAergic system that continues into early infancy [49]. Mid-late gestation sees a reorganization of GABA receptors and their inhibitory and excitatory balance, which is essential for brain development [26].

White matter (WM) is responsible for inter-neuronal connections throughout the brain that are a driving force in self-regulatory development. The association between the development of WM in early life and maturation of brain functioning as detected by EEG has been reported earlier [23,54,55]. In preterm infants born as early as mid-gestation, beyond the risk for destructive lesions, disruption of the normal developmental trajectory of cellular elements of the white and gray matter occurs. In the acute phase, in response to hypoxia–ischemia and/or infection and inflammation, multifocal areas of necrosis within the periventricular white matter involve all cellular elements. White matter injury, recognized as a major global health issue, is a well-known adverse outcome of extreme prematurity that disrupts the normal development of myelination [56].

The rapid changes in voltage- and transmission gated in a preprogrammed sequence differentiate the developing brain from the adult brain. This occurs through the creation of the Na-K pump in the neuron cell operating to determine the firing time and the recovery time of the cell. Intrinsic currents are followed by non-synaptic calcium plateau and synaptic depolarization potentials [57]. These cellular processes are mediated by signaling of GABA and N-Methyl-D-aspartic acid (NMDA) receptor currents including the removement of Mg+ voltage dependent blockade [57]. Synaptic burst activity leads to weak disorganized connectivity [58]. However, at the time behavior is coupled with these currents by mid-gestation, a preprogrammed “stop-signal” for the burst activity is apparent by the voltage-gated K+ signaling. Thereafter, “senior” older neurons wire together to create networks with other “senior” neurons” occurring earlier in older structures such as the brainstem and spinal cord than in “younger” structures [59,60,61]. Segregation and integration of information exists as early as the late stages of the second semester of gestation and the progress of their consolidation starts earlier at mid-gestation increasing with GA [62].

Additionally, the period from 23 to 32 weeks gestation constitutes the highest risk for white matter injury, and dysmaturation of WM [54] with a peak at 28 weeks’ gestation [55]. The complex network of structural connections that arise in this period shapes functional connectivity and higher order self-regulatory functions [59,63]. Specifically, this time window, validated in healthy fetuses, appears to be a critical period in brain development. MRI- Diffusion Tensor Imaging (DTI) studies focusing on the end of the second trimester, reported that for all tracts, the volume and fractional anisotropy (FA) increased, and the apparent diffusion coefficient (ADC) decreased with GA, though these associations were not statistically significant in all tracts [60,64]. The development of network connectivity, which starts in early gestation and accelerates in mid-gestation, continues over the third trimester and into the postnatal period, as shown by MRI studies (e.g., [61]).

Thus, the same time window of critical development from mid-gestation onwards is suggested for neurogenesis of WM and vagal myelination as well as for the changes in biochemical inhibitory and excitatory terminals in a clock-wise manner.

Additionally, during this time window of mid to late gestation, starting at 26 weeks GA and maturing at 38 weeks GA, the association between neurobehavioral measures and heart rate becomes more pronounced. Fetuses respond to stimulation, as measured by these two levels of functionality [65,66]. Vagal tone then becomes the main representation of the progressing maturation of ANS coupling with the developing cortex [67]. These two facets, neurobehavior and heart rate, show a chronological timewise development including the interactions and the coupling between them in a gradual process. Before 32 weeks GA, only periods of fetal activity and quiescence can be distinguished, while after 32 weeks four distinctive states can be discerned. The increasing synchronization of fetal movement and vagal tone reflects the development of regulation by the ANS [68,69,70], i.e., the developing acquisition of ANS synchronicity. Vagal myelination occurs during the entire period of term pregnancy and continues postnatally with hypo- and hyper-myelinated fibers. Unmyelinated fibers exist postnatally for up to one year of life [71]. Normal development of vagal myelination has been shown to be associated with improved self-regulation up to school-age [72].

### 3.3. Birth and Postnatal Period

Cortical regulation of the brainstem and coupling of the ANS with the cortex can be detected in changes in EEG waves from birth through early childhood. To continue the description of ANS synchrony developmental milestones beyond gestation, the main developmental pathways of the EEG waves that were found to be related to the emerging self-regulatory functions will be described.

In term born infants less than 2 months of age, periods of quiet sleep (or quiescence) are characterized by repetitive bursts of high-voltage slow wave electrical activity alternating with attenuated 3 Hz mixed-frequency activity. This characteristic pattern is known as Trace′-alternant (TA) [73,74]. TA is defined with 3–8 s bursts of high amplitude slow waves separated by 4–10 s low voltage mixed EEG (theta with some delta alpha and beta). This pattern disappears by 46–48 weeks GA and a more continuous sleep pattern appears during quiet sleep associated with higher heart rate variability [75].

Thus, the baseline EEG in newborn infants, measured with minimal or maximal number of electrodes and channels, is discontinuous. This pattern is characterized by bursts of high amplitude delta–theta activity (sometimes superimposed with faster activity) intermixed with periods of quiescence (interburst intervals—IBIs). With maturation of normal inhibitory GABAergic transmission, spontaneous events are gradually abolished and ‘continuous’ oscillations emerge at different frequencies due to the increasing influence of exogenous sensory driven input and more developed sensory sensitivity and gradual sensory integration. Consequently, the overall amount of discontinuity decreases, and continuity increases with GA as shown in comparisons of early and late born preterm infants. The EEG pattern that develops in the most immature newborns from 23–24 weeks through 40 weeks GA has four major trends: (i) increasing continuity, with defined periods of EEG quiescence for specific GAs, (ii) the appearance of sleep cycling, (iii) advances in synchrony between hemispheres, (iv) and the disappearance of several transient waveforms of prematurity [76]. Note that in the full-term newborn, an adjustment phase has been detected too. Specifically, TA has been identified postnatally by the time courses of quadratic phase-coupling of electroencephalographic burst and inter-burst patterns [77], suggesting another milestone in the developing regulatory clock discussed in this paper.

Sleep and wakefulness patterns mature through the inhibitory function of the post-synaptic GABAergic and cholinergic neuro-activity which interact to regulate the levels of excitatory norepinephrine and glutamate [78,79,80]. Inputs from the brainstem are modulated by the reticular formation, thalamus and the preoptic area of the hypothalamus on their pathways to the cortical circuits. GABAergic neurons are responsible for the timely impact on the hippocampal theta wave which characterizes early development, while cholinergic neurons are the pacemakers of the amplitude. In turn, glutamate acts as an excitatory end on the GABAergic and cholinergic neurons’ inhibitory function to support hippocampal theta rhythm. In addition, the number of spikes in a burst and the interburst frequency (2–14 Hz) are dependent on a consecutive regression in the brainstem cholinergic input [78,79,80]. Thus, the development of the continuous EEG patterns and especially, that of the first alpha and then the beta wave emergence from the theta-delta wave, promoting increasing low voltage fast activity of wakefulness and cortical functions, represents the development of the ANS synchrony that underlies the self-regulation clock. The emergence of brain synchronicity is in fact represented by the increased balance between excitatory and inhibitory terminals and developmental changes in the targeted receptors as well as mutual regulation between subcortical and cortical regions beyond the structural volumetric development and in parallel to the developing ANS synchronicity.

Furthermore, KCC2 is expressed around birth in the brainstem, then a week or two postnatally in the hippocampus and thereafter in the cortex [81,82]. Thus, while GABA is excitatory on immature neurons and has a trophic effect on cortical development, at least 20% of GABAergic neurons in the white matter migrate toward the cortex over late gestation. After term, migration declines and ends within 6 postnatal months [53].

The interchange between the excitatory sources, meaning shifting from excitatory acetylcholine and GABA activity to glutamatergic excitatory expression that becomes more dominant late in gestation, occurs within the time window of mid-gestation. This is also the time window for the GABA turnover to inhibitory function and for the increased vagal myelination towards brainstem maturation [31,53]. The regulated interchange between peaks of ACh and glutamate activity postnatally at ages of mid-gestation to term has a crucial role in affecting the developmental path of neural cell migration from the brainstem to the cortex [3], in term born infants. In addition, in term born infants, GABAergic granular neurons balance against excitatory (glutamatergic) pyramidal neurons that are required for self-regulation processing. This involves modulation of excitatory events by inhibitory neurons, as well as a coordinated balance between excitation and inhibition maintained over a large range for many stimuli.

Dysregulation in the peak interchange junction between excitatory and inhibitory neurotransmitters [26] may be the reason for the reduced/aberrant myelination of the vagus nerve in preterm infants exposed to the extra-uterine environment before term. The developmental progress in myelination [83] may play a crucial role in balancing the initiation of neural cell migration from early to late developing regions, thus supporting the emergence of self-regulatory functions in the neonate.

## 4. Brainstem Regulation of Cortical Development

The brainstem regulates the ANS coupling with the cortex [1] while ANS synchronization is dependent on cardiac development and its resulting effect on self-regulatory functions.

The brain-heart connection is a Darwinist proposition [84]. Indeed, the brain is connected to cardiac myocytes and arteriolar smooth muscle cells, which are the cardiovascular autonomic effectors, by di-synaptic neuronal connections of the preganglionic and ganglionic autonomic neurons [85]. It has been suggested that the expression of thalamocortical neurons, skeletal muscles, and the ANS, which may explain the EEG, electromyographic, and cardiovascular features are modulated and controlled by circuits in the brainstem [86], concomitantly with other regions. The cortical-ANS coupling may reflect the common dependence of EEG and heat rate variability (the vagus activity measurement) measures on the activity of brainstem circuits. The brainstem appears critical in this respect, as quintessential brainstem neural elements of autonomic, respiratory, and cardiovascular control are developing within the medulla, pons, and midbrain together with key elements of the stepwise progressive neuronal connectivity that control EEG activity through the thalamus and basal forebrain [86].

Vagal myelination progresses with age through the first months of postnatal life [87]. Developing cortical regions regulate the continuation of brainstem control of vagus myelination through the first months of life [88]. It has been argued that the vagal pulse represents the coupling of the heart with brain regions such as the medulla and frontocortical areas and that social interaction in early life is crucially supported by vagal myelination [89,90]. However, although the progress of vagal myelination is part of fetal development, these observations do not include embryonic spatiotemporal considerations nor a time scale of multi-system development as proposed in this paper. This paper describes, using a multi-system perspective, the embryonic phase to postnatal description of brainstem-cortex coupling, emphasizing the full spectrum of vagal progressive stages as its myelination rhythms are anatomically and biochemically reliant on—and conditional to—brainstem development.

With advanced cortical development, the cortex has greater control over the brainstem in direct (e.g., corticobulbar) and indirect (e.g., corticoreticular) pathways emerging in the motor cortex and ending in the source nuclei of the myelinated motor fibers that develop towards and from the brainstem [91,92]. This occurs at the end of healthy pregnancies through early childhood.

## 5. The Self-Regulation Clock

**Figure 5:**
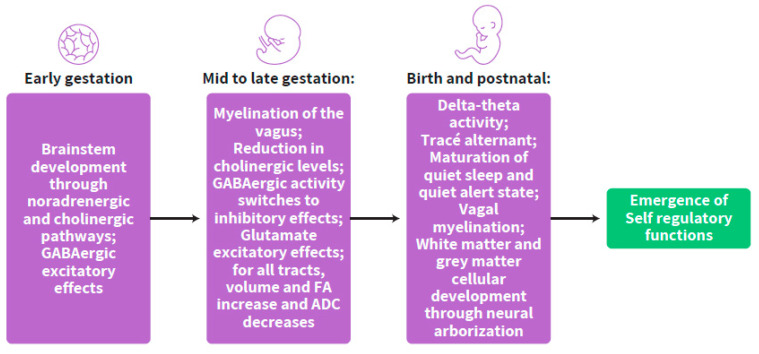
The stepwise developmental progress of the self-regulation clock from conception to postnatal life.

The changes in inhibitory and excitatory terminals during gestation from early to late stages are programmed in a stepwise timely manner, suggesting a clock for the maturation of ANS synchrony with a significant time window of massive changes at mid-gestation. In this regard, the prenatal advancing neurotransmitter interchange in the developing brain is suggested as a preparation of the fetus towards self-regulation in the extra-uterine environment. This preparatory phase is termed the “self-regulation clock” to be further developed after birth (Figure 5).

The self-regulatory clock milestones are viewed in this manuscript as the development of the synchrony between the sympathetic and parasympathetic systems and the developing control of the cortex over the brainstem with major implications for the emergence of germinal regulatory functions in the infant as the fundamentals of later to be developed self-regulatory behaviors. The development of regulatory functions follows a genetic clock with specific rhythms and time windows for each developmental acquisition prenatally and postnatally. 

At early gestation, the majority of the developmental processes that the embryo undergoes, both cellular and chemical, depend on sympathetic activity. By mid-gestation (23–25 weeks GA) the acetylcholine declines and the excitatory tone in the fetal brain turns to be dependent on glutamate; GABA turns to be inhibitory and not excitatory as earlier, and myelination of the vagus undergoes massive progression. This seems to be the time window for the generation of self-regulatory functions through the developing synchrony between the sympathetic and the parasympathetic systems although it has a germinal multi-level basis earlier in gestation. Here it is suggested that the self-regulatory clock is genetically programmed at an early gestational stage to reach the status of ANS synchrony. The dialogue of the growing infant with the environment by match-mismatch trajectories [93,94] may shape this clock up to the age of two years by which time the cortical-brainstem circuits should be established. There is much evidence to support the environment/experience’s influence on brain growth and neuronal communication in preterm infants. This is at the core of many developmental programs within the sensitive period of development, when an infant may require intensive care for survival. The self-regulation clock during mid-gestation is responsive to environmental inputs, as shown in intervention studies in preterm infants [19,54,95,96,97,98,99,100,101,102,103,104]. Thus, similarly to all biological clocks, the self-regulation clock is also sensitive to environmental cues be it the intra- or extra-uterine environmental inputs according to the timing of birth and the GA impacts.

Stress and disease may delay this accomplishment as multiple requirements are imposed on the organism with limited conditions for achievement of the clock trajectories aimed at synchronous functioning. The synchronous ANS maturation depends on the maturation of the connectivity between the brainstem and the cortex and the transition of control on self-regulatory functions from the brainstem to the cortex. Whereas global intact WM and development without neural insults is required for the emergence of optimal self-regulatory capacities in the newborn and preterm infant, it is proposed that the clock described here is an a-priori condition for these anticipated uncompromised regulatory functions and that this clock’s consolidation precedes the emergence of these behavioral functions as described in the literature on postnatal development. It is postulated here that the development of the self-regulation clock starts from early development of the brainstem and its onset occurs before the penetration of the thalamic fibers into the cortical plate and the resolution of the subplate [2]. It is further proposed here that the self-regulation clock is sensitive to environmental stimuli from early embryonic fetal and preterm infants’ life, and that neurobehavioral reactions appear at various levels of self-regulation in response to the given environment and a given GA, from mid-gestation onwards. The intra-individual perspective should be considered to “read” these early self-regulatory reactions as suggested earlier [8] to adjust the environmental stimuli to these behavioral signals and provide a proper “matching” rather than “mismatching” of the growing individual with the type and levels of external stimulation. This may have long lasting implications as it was shown recently to affect the transcriptome in early development and especially preterm infants who are born at the sensitive time window of mid-gestation [100,101,105].

### 5.1. Location of the Self-Regulation Clock

**Figure 6:**
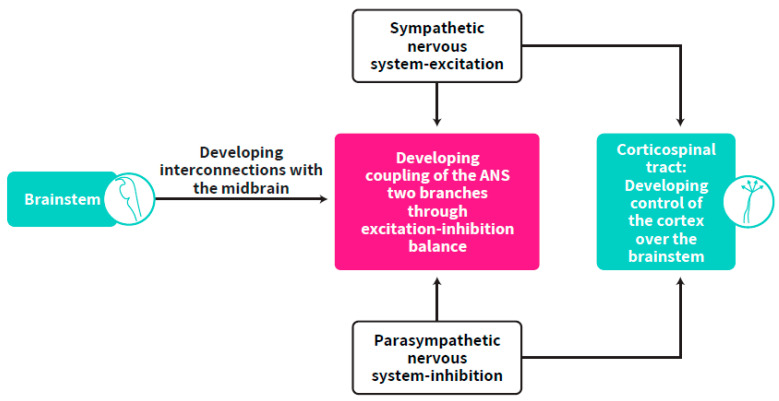
The origin of cortical control over the brainstem by which the CST develops to become the location of the self-regulation clock.

As both the sympathetic and parasympathetic systems originate in the brainstem and ANS synchrony is dependent on cortical-brainstem connectivity as well as on the developing control of the cortex over the brainstem, the self-regulation clock, which is dependent on ANS synchrony, is suggested to be located in the corticospinal tract (CST), the largest connection between the cortex and the brainstem. This developing structure, which is the basis for the maturation of cortical control over the brainstem, is responsible for cortex-brainstem connectivity (Figure 6). The CST is one of the pyramidal tracts, a white matter pathway starting at the cerebral cortex that terminates on lower motor neurons and interneurons in the spinal cord. Corticospinal tracts undergo earlier maturation than other tracts (as measured by fractional anisotropy) but slower volumetric growth, while the anterior to posterior gradient in white matter microstructure development found the posterior forceps developing at a faster rate than the anterior forceps minor between mid-gestation and two years of age [106]. This has implications for the time window for the development of cortical control over the brainstem, suggested as starting at mid-gestation and undergoing a long process up to one or two years of age while myelination increases through the first years of life [107]. After a few weeks of development, they progressively innervate the gray matter such that there is extensive innervation of spinal neurons, including motor neurons, prior to birth. Functional monosynaptic corticomotoneuronal projections were demonstrated neurophysiologically from term, but are also likely to be present from as early as 26 weeks GA [108]. After leaving the neocortex, CST axons form bundles and run through the internal capsule and cerebral peduncles before reaching the brainstem in a ventral position. CST axons maintain their ventral position (forming the pyramids) until they reach the caudal part of the medulla. At the junction between the brainstem and spinal cord, the vast majority of CST axons cross the midline and pass from a ventral to a dorsal position, forming the pyramidal decussation, before continuing their trajectory in the contralateral spinal cord [109,110]. The termination pattern of CST axons in the spinal gray matter depends on the cortical territory from which they originate, further stressing the divergent roles of this pathway [110].

Although the cortical plate is barely formed by 7 weeks GA, the CST axons reach the medulla by that time. These axons reach as far as the lumbar enlargement by 18 weeks GA and thereafter, the lower cervical spinal cord by 24 weeks GA. Following a waiting period of a few weeks, they progressively innervate the gray matter with a peak of the prolonged myelination progress at 40 weeks GA. Synaptic projections of the CST are established during the third trimester of pregnancy. It has been suggested that rather than furthering motor control, this massive development is aimed at coupling with the cortex and cortical development. Longitudinal and cross-sectional studies of normal babies and children report neurophysiological findings that are consistent with withdrawal in significant numbers of corticospinal axons over the first 24 postnatal months [111], providing evidence for continuous CST development into early childhood [107]. The final pattern of the origin and termination of the CST is shaped during development by the balance between projection and withdrawal of axons [105]. The time window for prenatal and postnatal CST development coincides with the same timing of massive fetal and newborn development, described here as the basis for the developing clock of self-regulation. This suggests that its myelination represents the chrono-progression of ANS coupling with the cortex through the brainstem. As the CST is the largest connection between the cortex and the brainstem and involved in transferring the control over to the brainstem as well as in the maintenance of ANS synchrony, it is suggested that the ontogenetic developing function of the self-regulation clock may be defined as the measurable ratio between the maintained balance within biochemical secretion of neurotransmitters and the gradients of CST volume development.

The early development of the CST during gestation and the corresponding neurogenesis progression with a mandatory crucial role in the developing control of the cortex over the brainstem and in ANS functionality maturation, suggests that this tract’s time window for development is vital for WM normal development and resulting neurobehavioral parameters such as self-regulation. Additionally, as described above, wiring towards the creation of networks starts in “older\senior” structures such as the brainstem and the spinal cord, which are pathways of the CST, and the advance in this network’s creation progresses from mid-gestation onwards.

Taken together, the ratio between the neurotransmitters’ interchange and the role of CST in cortex maturation through the impacts of developing control of the cortex over the brainstem while the brainstem generates the synchronization within the ANS, suggests that an independent ontogenetic clock of self-regulation is seated in CST function and develops in a stepwise ontogenetic manner with direct and indirect effects on the emergence of executive functions later in childhood [112,113,114], and that this clock is sensitive to the balance between neurotransmitters and levels of synaptic storage during gestation and especially after early birth.

### 5.2. Rhythms of the Clock

The suprachiasmatic nucleus (SCN) projects to presympathetic and to some preparasympathetic neurons as well as to peripheral oscillators including in the spinal cord, where the CST is ending [115]. The oscillatory functions start from a combination of signals and noise [116,117]. This reflects the coupling of the SCN and its central clock with ANS and CST. Both CST and SCN are partially active from fetal life onwards, and they mature postnatally [107,117]. The maturation of the SCN precedes that of the CST [27,107].

Our proposal regarding particular developmental rhythms and timing of self-regulatory milestones is further supported by other studies that showed the involvement of the CST in biological clocks, such as those responsible for diurnal rhythms [118,119]. It is well known that an infant is optimally available for human interaction which requires self-regulatory executive functions following feeding and organized sleep and this increases with age. As self-regulatory functions which affect the quality of the infant’s executive functioning are dependent on the ANS’ maturational status modulated by the developmental stage of the circadian rhythms in term and preterm infants [27], this may imply an additional role of the CST suggesting its involvement in regulating the day-night cycle through its coupling with cortical and sub-cortical regions. To support this view, others showed that greater age and improved state organization (sleep-wake cycle in newborns) are negatively associated with atypical corticospinal tract development in infants [120]. More broadly the fetus is spending the time growing in the womb mostly asleep up to mid-gestation (i.e., inhibition-control of the state subsystem) while increase in awake states occurs from mid-gestation onward (i.e., developing inhibition-excitation equilibrium) suggesting the same time window as noted earlier, mid-gestation, as a period of accelerated SCN and CST involvement in fetal diurnal rhythms beyond maternal melatonin impact via placental pathways as earlier thought. Later, after birth, the challenge of widening the range of quiet alert state (awake) is the first outcome of optimal self-regulation in the newborn (i.e., inhibition-excitation balance acquisitions). Thus, the view that the CST may be involved in the diurnal clock maturational process supports the view that this tract is also the location of the developing self-regulation clock through brainstem-cortex maturation and brainstem modulation of the emerging synchronization within the ANS. This may be the basis for new hypotheses on the involvement of classical sensorimotor regions such as the CST and their promoting function in the developing balance within the ANS including its role in the emergence of executive function.

In support, diurnal rhythms are apparent in the spinal reflex and somatosensory evoked potentials are entrained by the light-dark cycle, which suggests that they may be coupled to the same oscillator [121]. Circadian rhythms regulated by the CST have been shown in animals using the H-reflex [122]. As the H-reflex is dependent on gravity, the earlier experience of the sense of gravity in preterm infants (when they emerge from the amniotic fluid) may alter the diurnal clock and diurnal H-reflex that is modulated by spinal reflexes [123], while the spinal cord is the location of CST terminals. This alteration in spinal-controlled diurnal rhythms has been related to synaptic dysregulation in neurotransmitters’ functions [124], supporting our earlier arguments. The scarce available data on SCN-CST interconnectivity suggests only indirect connections and projections between SCN and CST but nevertheless mutual and associated reactivity of the two structures as a part of a yet unknown complex circuitry involving the coupling of rhythms with behavior during early development. Further research on these indirect projections and pathways between SCN and CST is warranted, especially on the two structures’ early trajectories during the time windows highlighted here, to compare healthy to compromised growth and their dependency on GA.

## 6. For Further Research

As a starting point of a research endeavor, the association between the volume (and function) of the CST and the scores in self-regulation tests such as the Assessment of Preterm Infants’ Behavior (APIB), The Brazelton test and the Prechtl General Movements Assessments should be tested to critically examine the hypothesis that self-regulation is part of (or at least strongly associated with) CST functions. Further steps in research may need to follow up on various stages of the CST development with imaging and the Fetal Finger Index, which has been shown to predict neurobehavioral outcomes after birth [125]. On the molecular level, self-regulation, described by a balanced ratio between acetylcholine and cortisol postnatally, is suggested as a research target to be correlated with CST volume and function. At an advanced stage, ivestigations whether interventions in Neonatal Intensive Care Units that promote self-regulation within the time windows detailed in this paper, will increase the volume of the CST and its connectivity as outcome measures in preterm infants, are warranted. In addition, preterm infants who suffer from asphyxia during the window of opportunities for the maturation of the CST as detailed in this paper, could be tested for size and connectivity of the CST as well as for the association of CST connectivity with the CA+K+ pump which, in turn, is known to collapse during asphyxia [126].

## 7. Conclusions

The hypothesis presented here is based on the development timeline of corticospinal tracts. However, it does not exclude the development of other associations to fiber tracts that occur in a similar timeline. Finally, the excitation-inhibition balance of sympathetic and parasympathetic activity was defined as the key measure for ANS maturation. However, it is important to note that these processes are not antagonistic and they work in a developing synergy in healthy fetuses similar to their synergic action in inflammation (e.g., [127,128]).

The concept of self-regulation has been postulated to underlie later developmental age-appropriate accomplishments of the infant, including cognitive capacities [129]. This concept has not been related to an overarching outline of ANS synchrony and its developmental pathway from fetal life to early childhood as yet. Self-regulation has been mostly measured behaviorally. This paper suggests that ANS synchrony represents the biological basis for the developing self-regulatory functions, here termed the self-regulation clock. This is the first review to propose a developmental process of the ANS, which seeks balance and is suggested as a prerequisite for the emergence of future optimal development. The synchronization between the two branches of the ANS is sensitive to internal tonic and phasic crosstalk and to external level of input. It is suggested that the key role of ANS maturation follows a stepwise ontogenetic clock. This clock is apparent in the neuron cell, neurotransmitter interchange, WM development, vagal myelination and vagal activity and maturation of EEG patterns. Multiple types of data show accumulating evidence of the time windows of this stepwise process emphasizing the association between optimal development and optimal outcome. The initiation of the self-regulation clock early during gestation is continued in a complex multi-level progression in an excitation-inhibition manner. It starts from early brainstem development, in the development of the sympathetic and parasympathetic systems and progresses to a complex development of cortical control over the brainstem through the largest connection pathway, the CST. The processes described in this article progress from early gestation up to two years postnatally. Our evidence-based theorem paves the way towards important hypotheses and studies on the self-regulation clock, which is central to normal development and comprises the time windows for interventions to promote preterm infants’ neurobehavioral functions, which in turn are essential for these infants’ healthy survival.
